# The distribution and reliability of TMS-evoked short- and long-latency afferent interactions

**DOI:** 10.1371/journal.pone.0260663

**Published:** 2021-12-14

**Authors:** Stephen L. Toepp, Claudia V. Turco, Ravjot S. Rehsi, Aimee J. Nelson

**Affiliations:** Department of Kinesiology, McMaster University, Hamilton, Ontario, Canada; BG-Universitatsklinikum Bergmannsheil, Ruhr-Universitat Bochum, GERMANY

## Abstract

Short-latency afferent inhibition (SAI) and long-latency afferent inhibition (LAI) occur when the motor evoked potential (MEP) elicited by transcranial magnetic stimulation (TMS) is reduced by the delivery of a preceding peripheral nerve stimulus. The intra-individual variability in SAI and LAI is considerable, and the influence of sample demographics (e.g., age and biological sex) and testing context (e.g., time of day) is not clear. There are also no established normative values for these measures, and their reliability varies from study-to-study. To address these issues and facilitate the interpretation of SAI and LAI research, we pooled data from studies published by our lab between 2014 and 2020 and performed several retrospective analyses. Patterns in the depth of inhibition with respect to age, biological sex and time of testing were investigated, and the relative reliability of measurements from studies with repeated baseline SAI and LAI assessments was examined. Normative SAI and LAI values with respect to the mean and standard deviation were also calculated. Our data show no relationship between the depth of inhibition for SAI and LAI with either time of day or age. Further, there was no significant difference in SAI or LAI between males and females. Intra-class correlation coefficients (ICC) for repeated measurements of SAI and LAI ranged from moderate (ICC = 0.526) to strong (ICC = 0.881). The mean value of SAI was 0.71 ± 0.27 and the mean value of LAI was 0.61 ± 0.34. This retrospective study provides normative values, reliability estimates, and an exploration of demographic and testing influences on these measures as assessed in our lab. To further facilitate the interpretation of SAI and LAI data, similar studies should be performed by other labs that use these measures.

## Introduction

Afferent inhibition is observed when the motor evoked potential (MEP) elicited by transcranial magnetic brain stimulation (TMS) is supressed by a preceding peripheral nerve stimulus. This phenomenon may be observed when nerve stimuli are delivered at long (200-1000ms) or short (20-25ms) inter-stimulus intervals (ISI) with respect to the subsequent TMS pulse [1–3]. Cholinergic activity has been shown to modulate short-latency afferent inhibition (SAI), which is also suppressed in cognitive disorders [4–7]. Both SAI and afferent inhibition elicited at long ISIs (200-1000ms; LAI) are reduced by positive allosteric modulators of the gamma-aminobutyric acid Type A (GABA_A_) receptor. Both LAI and SAI are reduced (i.e., less inhibition) in a wide range of special populations compared to healthy controls, including disorders of cognition [5, 8, 9] and diseases affecting the sensorimotor system such as Parkinson’s [10, 11] and amyotrophic lateral sclerosis [12]. However, much remains unclear about the underlying neurophysiology of afferent inhibition. The fact that inhibition occurs at different ISIs for SAI versus LAI indicates that distinct pathways are traversed by the afferent volley in inhibiting the MEP, but a definitive profile of the specific circuitries underpinning each measure remains elusive [2]. Further, behavioural correlates are not well-established for either SAI or LAI [13].

Efforts to resolve these questions and further advance the clinical utility of SAI and LAI have been hampered by their low-to-moderate relative reliability and high levels of measurement error [14, 15]. Importantly, in our experience it is not uncommon for an experienced researcher to fail in eliciting SAI and/or LAI in a healthy test subject with no observable neurological abnormality. Further, study participants for whom inhibition is observed during one testing session may not express inhibition on a separate occasion despite no notable change in behavior or health status (e.g., Turco et al., [14]). It is therefore important to establish the reliability of these measures across larger sample sizes to properly interpret study results.

In this retrospective study, we profile the reliability of SAI and LAI measurements recorded in studies published by our lab since 2014 that included repeated baseline measures and provide normative values for SAI and LAI in healthy participants. We also present data from each study and investigate overall patterns in the frequency and depth of inhibition with respect to age, biological sex, and time of day.

## Methods

### Subjects

Data from healthy control participants (n = 170, 73 female) from eight studies that were conducted between 2014 and 2020 in the Neurophysiology and Imaging Laboratory (McMaster University, Hamilton ON, Canada) were included in this retrospective study [14, 16–22]. Studies and participant demographics are displayed in Table 1. All studies received approval from their corresponding local ethics boards (Hamilton Integrated Research Ethics Board; McMaster Research Ethics Board), and all participants provided informed written consent. All studies were conducted in accordance with the principles of the declaration of Helsinki.

**Table 1:** Participant demographics.

Study	N (F/M)	Age (years)
**A**	18 (11/7)	20.94 ± 1.98
**B**	12 (8/4)	20.91 ± 2.87
**C**	23 (Exp 1; 13/10)	23 ± 1.5 (Exp 1)
**D**	20 (15/5)	23.4 ± 5.2
**E**	14 (0/14)	22.7 ± 1.9
**F**	30 (15/15)	20.9 ± 2.5
**G**	18 (0/18)	22.8 ± 2.4
**H**	35 (35/0)	59 ± 3

A = Tsang et al. 2014; B = Tsang et al. 2015; C = Bailey et al. 2016; D = Turco et al. 2017; E = Turco et al. 2018; F = Turco et al. 2019; G = Toepp et al. 2019; H = Harasym et al. 2020.

### LAI and SAI acquisition protocols

The specific procedures used for assessing SAI and LAI can be found in Table 2.

**Table 2:** Study parameters and rules for data extraction.

Study	TMS	Nerve Stimulation	ISI	Experimental design	Origin of dataset 1	Origin of dataset 2
**A**	Target muscle: right FDI	MN stimulation at wrist set to motor threshold of APB muscle	SAI: N20+5ms	Exp 1: SAI assessed before/after cTBS to M1 or S1 in 2 separate sessions	Baseline SAI from first session	Baseline data from both sessions
	Intensity: 1mV MEP					
	20 conditioned, 20 unconditioned stimuli					
**B**	Target muscle: right APB	MN stimulation at wrist set to motor threshold of APB muscle	SAI: N20+5	Exp 1: SAI assessed before/after rPAS to M1 or S1 in 2 separate sessions	Baseline SAI from first session	Baseline data from both sessions
	Intensity: 1mV MEP					
	15 conditioned, 15 unconditioned stimuli					
**C**	Target muscle: right FCR	MN stimulation at elbow set to 50% SNAP_max_	SAI: N20+2ms	Exp 1: SAI assessed at one timepoint on one session	All SAI data used	N/A
	Intensity: 1mV MEP					
	15 conditioned, 15 unconditioned stimuli					
**D**	Target muscle: right FDI and APB	MN stimulation at wrist set to motor threshold of APB muscle	LAI: 200ms	Exp 1: LAI assessed at one timepoint on one session	LAI averaged across target muscles	N/A
	Intensity: 1mV MEP					
	15 conditioned, 15 unconditioned stimuli					
**E**	Target muscle: right FDI	MN stimulation at wrist set to motor threshold of APB muscle	SAI: N20+4ms or N20+6ms	Exp 1: SAI/LAI assessed before/after administration of lorazepam, baclofen or placebo in 3 separate sessions	SAI and LAI averaged across ISIs	SAI and LAI averaged across ISIs and for each ISI
	Intensity: 1mV MEP					
	15 conditioned, 15 unconditioned stimuli		LAI: 200, 400, 600ms			
					Baseline SAI/LAI from first session	
						Baseline data from all 3 sessions
**F**	Target muscle: right FDI	MN stimulation at wrist set to motor threshold of APB muscle	SAI: N20+4ms or 24ms	SAI/LAI assessed in 2 separate sessions	SAI averaged across ISIs	SAI averaged across ISIs and for each ISI
	Intensity: 1mV MEP					
			LAI: 200ms			
	12 conditioned, 12 unconditioned stimuli				SAI/LAI data from first session	
						SAI/LAI data from both sessions
**G**	Target muscle: right FDI	MN stimulation at wrist set to motor threshold of APB muscle	SAI: N20+4ms	SAI/LAI assessed before/after administration of water, glucose or sucralose drink in 3 separate sessions	Baseline SAI/LAI from first session	Baseline data from all 3 sessions
	Intensity: 1mV MEP		LAI: 200ms			
	12 conditioned, 12 unconditioned stimuli					
**H**	Target muscle: right APB	MN stimulation at wrist set to motor threshold of APB muscle	SAI: 24ms	SAI/LAI assessed at one timepoint on one session	All SAI data used	N/A
			LAI: 200ms			
	Intensity: 1mV MEP					
	15 conditioned, 15 unconditioned stimuli					

Study Legend: A = Tsang et al. 2014; B = Tsang et al. 2015; C = Bailey et al. 2016; D = Turco et al. 2017; E = Turco et al. 2018; F = Turco et al. 2019; G = Toepp et al. 2019; H = Harasym et al. 2020. Abbreviations: SAI = short-latency afferent inhibition, LAI = long-latency afferent inhibition, ISI = inter-stimulus interval, APB = abductor pollucis brevis, FDI = first dorsal interosseous, FCR = flexor carpi radialis, MEP = motor evoked potential, MN = median nerve, N/A = not applicable.

### Data processing and statistical analysis

#### Main analyses

The dataset included in main analyses was comprised of the first acquisition of SAI and/or LAI data collected from each tested participant. Only data from right-handed participants were included. In studies where SAI or LAI were assessed using multiple ISIs, the data was averaged across ISI. One-way Kruskal-Wallis tests were performed to assesses for the effect of study on SAI (7 studies), and LAI (5 studies) values. These analyses were performed on SAI and LAI ratios of the average conditioned MEP to the average unconditioned MEP (MEP_CSTS_ /MEP_TS_). Spearman’s rho was computed to assess the relationships between age and the depth of SAI and LAI for participants between the ages of 18 and 35 years, as insufficient data was available for participants above the age of 35 years. In addition, the relationship between time of day and depth of SAI/LAI was assessed with Spearman’s rho. To test for the presence of an effect of biological sex on SAI and LAI, a nonparametric Mann-Whitney U test was performed comparing the depth of SAI/LAI in males versus females.

SAI and LAI observations were separated into bins (width of 0.2 with respect to the MEP_CSTS_ /MEP_TS_ ratio) to visualize the distribution of ratios across participants. Further, these bins were separated into the mean MEP_TS_ and MEP_CSTS_ values to confirm that there was significant inhibition within each bin. To do so, agreement of two normality tests (D’Agostino-Pearson test and Shapiro-Wilk test) was required to establish that the data within each bin did not violate the assumption of normality. In cases where this criterion was satisfied, one-sided paired t-tests were performed comparing the mean MEP_TS_ and MEP_CSTS_. Otherwise, a non-parametric Wilcoxon’s signed-rank test was performed to compare the mean MEP_TS_ and MEP_CSTS_. Bonferroni corrections were applied to adjust the alpha level based on the number of comparisons performed on the same dependent variable (i.e., SAI or LAI). Comparisons were only performed within bins where there were more than 8 observations which is the minimum to assess normality using D’Agostino-Pearson test (i.e., the normality test with the fewest required observations).

#### Reliability and reproducibility

Reliability statistics were assessed for studies with repeated baseline assessments of SAI and LAI. Initially all data were included in the analysis, irrespective of the inclusion/exclusion criteria of the individual studies. These data were then checked for outliers using Grubb’s test. Reliability was assessed according to study and according to the ISI used for SAI and LAI measurements. That is, when SAI or LAI was assessed using multiple ISIs within the same study, statistics were calculated using the average measure across ISIs, and also calculated using the values obtained at each independent ISI. ICCs were computed to determine the relative reliability of data within the included studies. A two-way random effects model ICC (2, k) was used, and 95% confidence intervals (CI) were reported with coefficients of variation (CV): CV = SD/mean×100. Since Study F included these analyses at ISIs of 24ms and N20+4 in the originally published report [14], those results are referenced in this work in lieu of repeating the analysis. The following cut-offs were used to interpret the ICCs in accordance with both Koo and Li [23] and Portney and Watkins, [24]; >0.9 indicates excellent relative reliability, 0.75–0.9 is strong, 0.5–0.75 is moderate, < 0.5 is low.

To further investigate the between-session variability in SAI and LAI, the mean absolute between-session difference was calculated for each participant that had multiple baseline datasets. When there were only two sessions, the absolute value of the between-session difference was taken. A Kruskal-Wallis test was used to assess potential variation in between-session differences between the included sessions. To explore effects of TS intensity on SAI and LAI variability, between-session differences when the TS intensity was above versus below 120% of RMT were compared using a Mann Whitney U test (two-tailed), and Spearman’s test was used to assess correlations of TS intensity (including above and below 120% RMT) with between-session differences in SAI or LAI. Repeated SAI and LAI measurements, were also grouped according to whether the measures crossed between inhibition and facilitation, maintained facilitation, or maintained inhibition. When the participants were assessed using multiple ISIs (i.e., for Studies E and F), each ISI was considered as an independent case. Between-session differences were then calculated using the log-transformed SAI and LAI ratios.

## Results

### Distribution of SAI data

**Fig 1:**
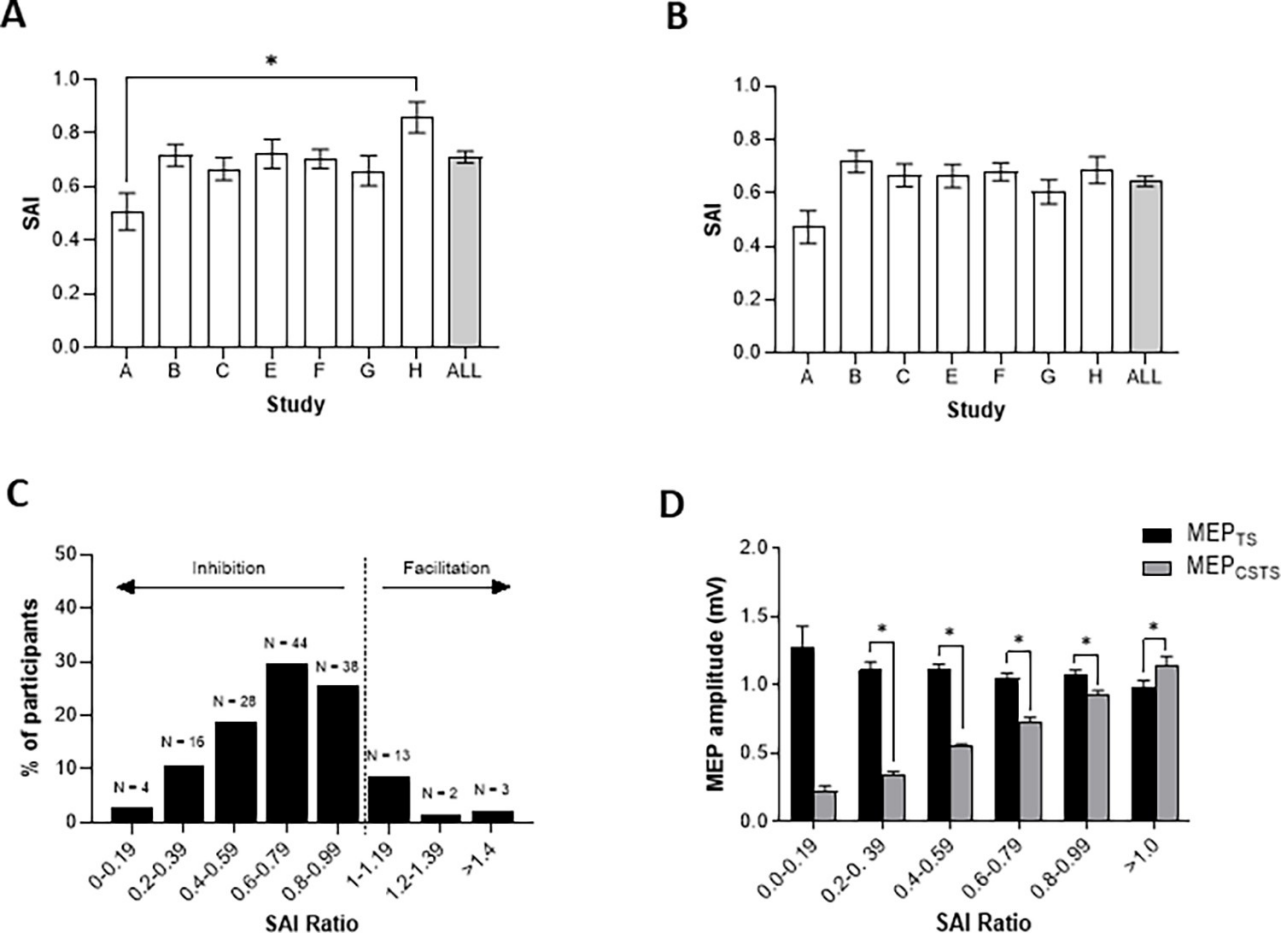
Distribution of SAI data for individual studies and data from all studies. SAI values from each of the studies included in the main analyses are shown with (A; n = 148) and without (B; n = 130) instances where no inhibition (ratios >1) occurred. The distribution of pooled SAI data (n = 148) is shown in C, and MEP_CSTS_ (gray) and MEP_TS_ (black) amplitudes are shown in D (also n = 148) for 20% bins between SAI = 0 and SAI = 1.0, and for SAI > 1.0. * p < 0.01 significant result of a Mann-Whitney U post-hoc comparison between study A and study H (A). * p < 0.01 significant difference indicated by Wilcoxon signed rank tests and for paired comparisons of MEP_CSTS_ and MEP_TS_ amplitudes within each bin (D). All error bars represent the mean and standard error of the mean.

Assessments of SAI (n = 148) were obtained from seven studies. Fig 1A shows the group-averaged means for all SAI data showing a main effect of STUDY (H(6) = 19.603; p = 0.003), whereby SAI is stronger (i.e., greater inhibition) in Study A compared to Study H (p < 0.001). The average SAI (± standard deviation) across all studies was 0.71 ± 0.27 (i.e., ~30% inhibition). The data in Fig 1B includes only observations where SAI was less than 1.0 (i.e., 18 observations where inhibition was not elicited were removed). Following removal of these data, a Kruskal-Wallis test indicated a main effect of STUDY (H(6) = 12.956; p = 0.044), but Bonferroni-corrected post-hoc comparisons (significance: p < 0.002) were no longer significant (p = 0.004). The average SAI following the removal of these data was 0.64 ± 0.21 (i.e., ~36% inhibition). To visually explore the depth of SAI, data were grouped into bins of 0.2. As can be seen in Fig 1C, ~3% of individuals demonstrate strong inhibition with ratios of 0.0 to 0.19 (i.e., 81% to 100% inhibition), 85% of individuals show ratios between 0.2 to 0.99 (i.e., 80% to 1% inhibition), while no inhibition is observed in ~12% of individuals (i.e., ratio >1). To confirm that the unconditioned MEP was different from the conditioned MEP in each of the bins shown in Fig 1C, each individual MEP_TS_ was compared to the MEP_CSTS_ and is shown in Fig 1D. Significant inhibition (p < 0.01) was observed for all SAI bins between 0.2 and 0.99. Significant *facilitation* was observed for ratios greater than or equal to 1.0 (p < 0.01). There were no differences between MEP_TS_ amplitudes across bins (H(6) = 7.089; p = 0.214).

### Distribution of LAI data

**Fig 2:**
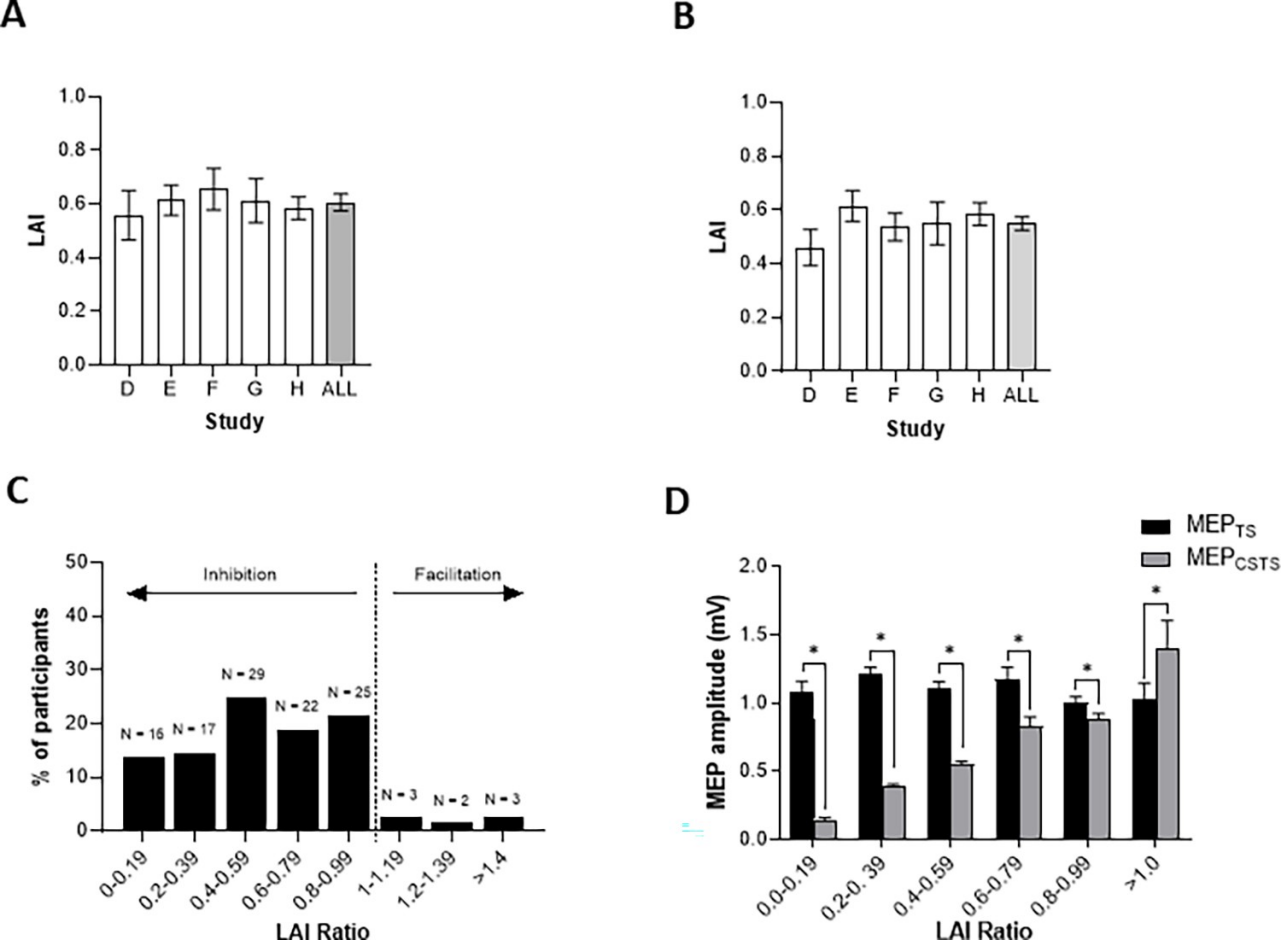
Distribution of LAI data for individual studies and data from all studies. LAI values from each of the studies included in the main analyses are shown with (A; n = 117) and without (B; n = 108) instances where no inhibition occurred. The distribution of pooled LAI data (n = 117) is shown in C, and MEP_CSTS_ and MEP_TS_ amplitudes are shown in D (also n = 117) for 20% bins between LAI = 0 and LAI = 1.0, and for LAI > 1.0. * p < 0.01 significant result of a Mann-Whitney U post-hoc comparison between study A and study H (A). * p < 0.01 significant difference indicated by Wilcoxon signed rank tests and for paired comparisons of MEP_CSTS_ and MEP_TS_ amplitudes within each bin (D). All error bars represent the mean and standard error of the mean.

Fig 2A displays the group-averaged means for all LAI data, revealing no significant difference across studies (H(4) = 1.25; p = 0.870). The average LAI across all studies is 0.61 ± 0.34 (~40% inhibition). Inhibition is not observed in 7% of individuals (8/117) and when removed does not impact the statistical outcome (H(4) = 3.189; p = 0.527; Fig 2B) with the average LAI equal to 0.55 ± 0.26 (~45% inhibition). The data are grouped into increasing bins of 0.2 MEP_CSTS_/MEP_TS_ (Fig 2C). Observation frequencies are relatively consistent for LAI ratios less than 1.0 with 14% to 24% of observations falling within each bin. Only 8 LAI values (~7%) exceeded 1, compared to 18 for SAI (~12%). To confirm that the unconditioned and conditioned MEP were different in each bin, we compared the individual MEP_TS_ and MEP_CSTS_ amplitude within each bin as shown in Fig 2D. Paired comparisons revealed significant inhibition for all LAI bins between 0.0 and 0.99 (all p < 0.01). There was no significant effect of bin on MEP_TS_ (H(5) = 10.77; p = 0.056).

### Association of SAI and LAI with age, time of day, and biological sex

**Fig 3:**
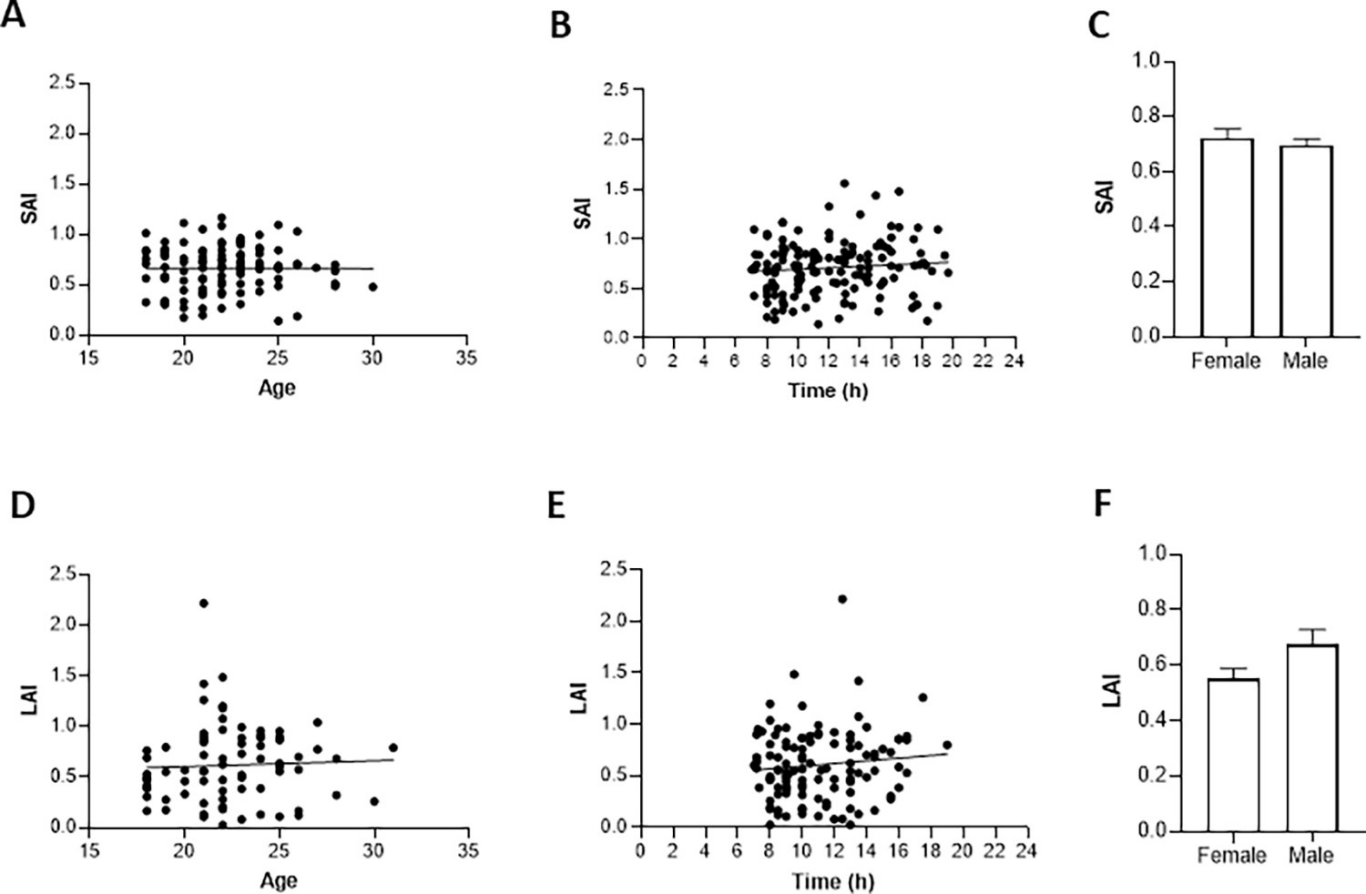
Correlations and gender comparisons for SAI and LAI data. Nonsignificant correlations of SAI with age (18–35 years; n = 113), and time of day (n = 148) are shown in A and B respectively. No difference in SAI with respect to biological sex is shown in C. Similarly, LAI did not correlate with age between 18 and 35 years (D; n = 81), or time of day (E; n = 117) and was not different between males and females (F). All error bars reflect standard error of the mean.

No significant correlations were observed between the depth of inhibition for SAI and both age (rho = -0.004, p = 0.973; Fig 3A) and time of day (rho = 0.089, p = 0.280; Fig 3B). SAI from males and females is represented with mean and standard error in Fig 3C. The mean difference between the sexes was not significant (p = 0.450; Fig 3C).

For LAI, no significant correlation was detected between the depth of inhibition and either age (rho = 0.096, p > 0.392; Fig 3D) or time of day (rho = 0.068, p = 0.465; Fig 3E). The difference between male and female LAI measurements was not significant (p = 0.070, Fig 3F).

### Between-session differences

**Fig 4:**
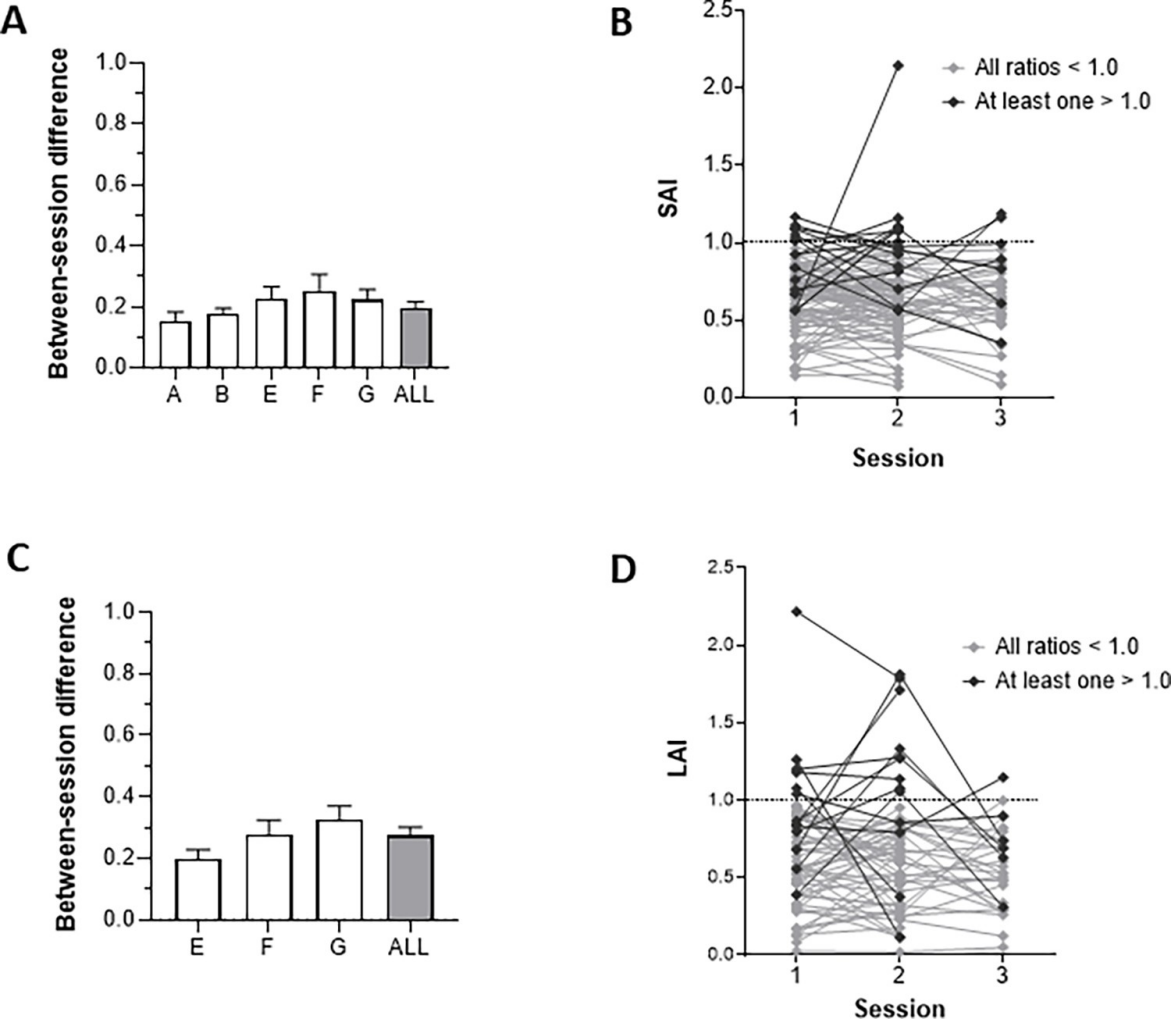
Reproducibility of SAI and LAI. The absolute size of between-session differences for studies with repeated baseline measures are shown on the left and individual SAI and LAI values are shown on the right. Between-session differences in SAI for each study (A; n = 92) are represented in the open bars while the average across all included studies is shown by the gray bar. Individual SAI data from the same studies is presented in B. Subjects who exhibited at least one SAI value > 1 are highlighted in dark gray while the rest are shown in light gray. Between-session differences in LAI for each study (C; n = 62) are represented in the open bars while the average across all studies is shown by the gray bar. Individual LAI data from the same studies is presented in D. All error bars reflect standard error of the mean.

The mean difference between sessions for individuals in whom SAI was tested on multiple days is shown for each study and averaged across all studies in Fig 4A. There was no effect of STUDY (H(5) = 3.02; p = 0.554) and the mean between-session difference in SAI across all studies was 0.21 ± 0.22. Likewise, there was also no effect of STUDY for between-session LAI differences (H(3) = 3.66; p = 0.161; Fig 4C), and the mean across all studies was 0.27 ± 0.22. Individual measurements for SAI and LAI are shown in Fig 4B and 4D, respectively. Seventeen percent of individuals (16 of 92) showed SAI > 1.0 on at least one occasion, whereas 21% (13 of 62) exhibited LAI > 1.0 at least once.

**Fig 5:**
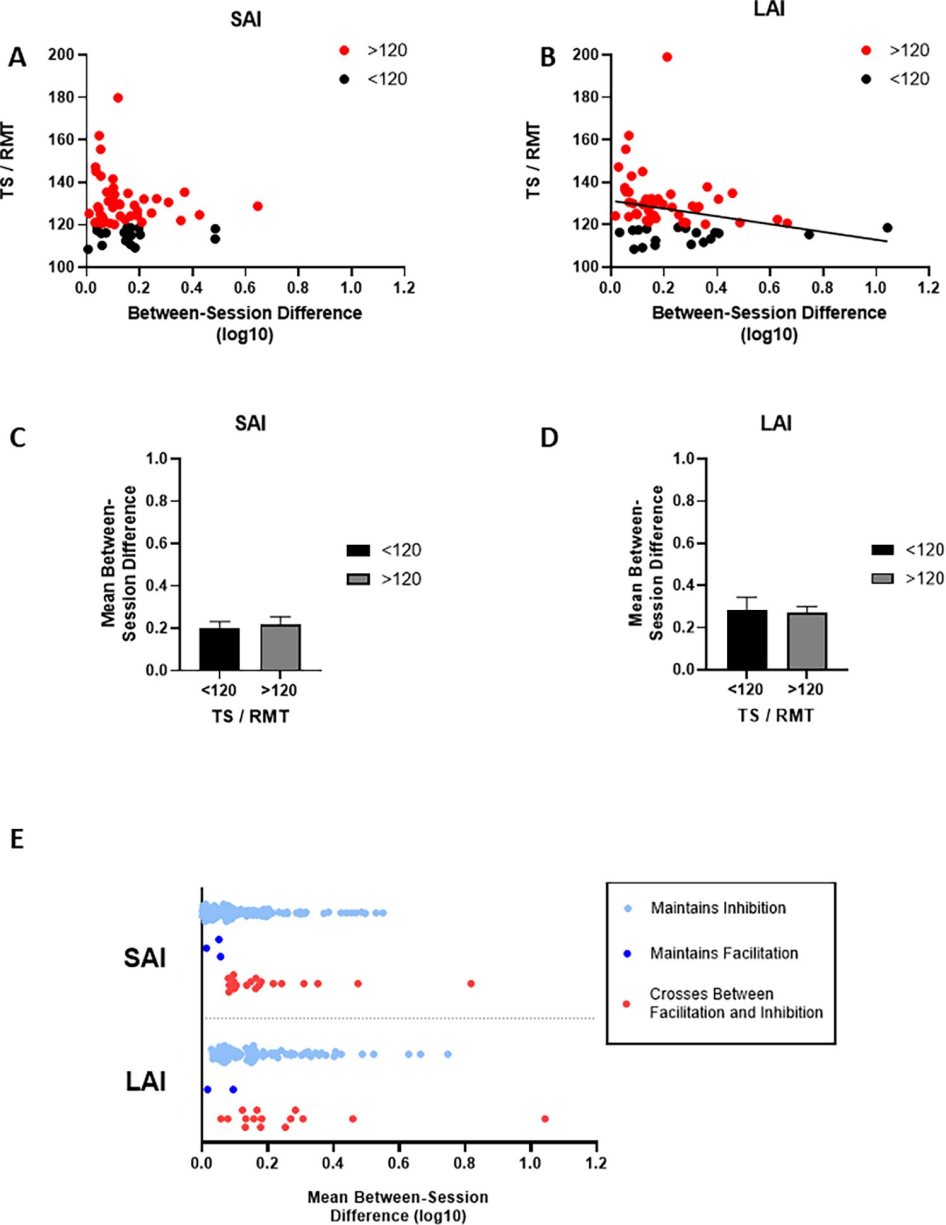
TS Intensity and inhibition-facilitation fluctuations. Panel A and B show the association between test stimulus intensity and between-session differences for log-transformed SAI and LAI, respectively. In panel C and D, between-session differences when the mean TS intensity was >120%RMT versus <120%RMT is shown for SAI and LAI, respectively. Panel E shows between-session differences in log transformed SAI and LAI ratios, separated into cases where participants always exhibited inhibition (light blue), always showed facilitation (dark blue), or crossed between inhibition and facilitation (red). Participants from Study E and F, who were assessed at multiple ISIs for SAI and/or LAI appear on the plot once for each assessed ISI.

Fig 5 shows the results of analyses concerning the association between TS intensity and between-session variability. No association was found between the average TS intensity and between-session differences (p = 0.126; Fig 5A), but TS intensity was negatively associated with between-session differences in LAI (rho = -0.394, p = 0.002; Fig 5B). The size of between-session fluctuations for TS intensities above 120% of RMT was similar to that when TS intensities were lower than 120% of RMT for SAI (p = 0.839) and LAI (p = 0.871). Fig 5E plots the between-session differences for instances whereby participants always demonstrated facilitation (dark blue), inhibition (light blue) and when participants crossed between facilitation and inhibition (red). With the exception on one outlier for each circuit, these data suggest that the between-session variability is similar between those who consistently show inhibition and those that fluctuate between inhibition and facilitation.

### Reliability

**Fig 6:**
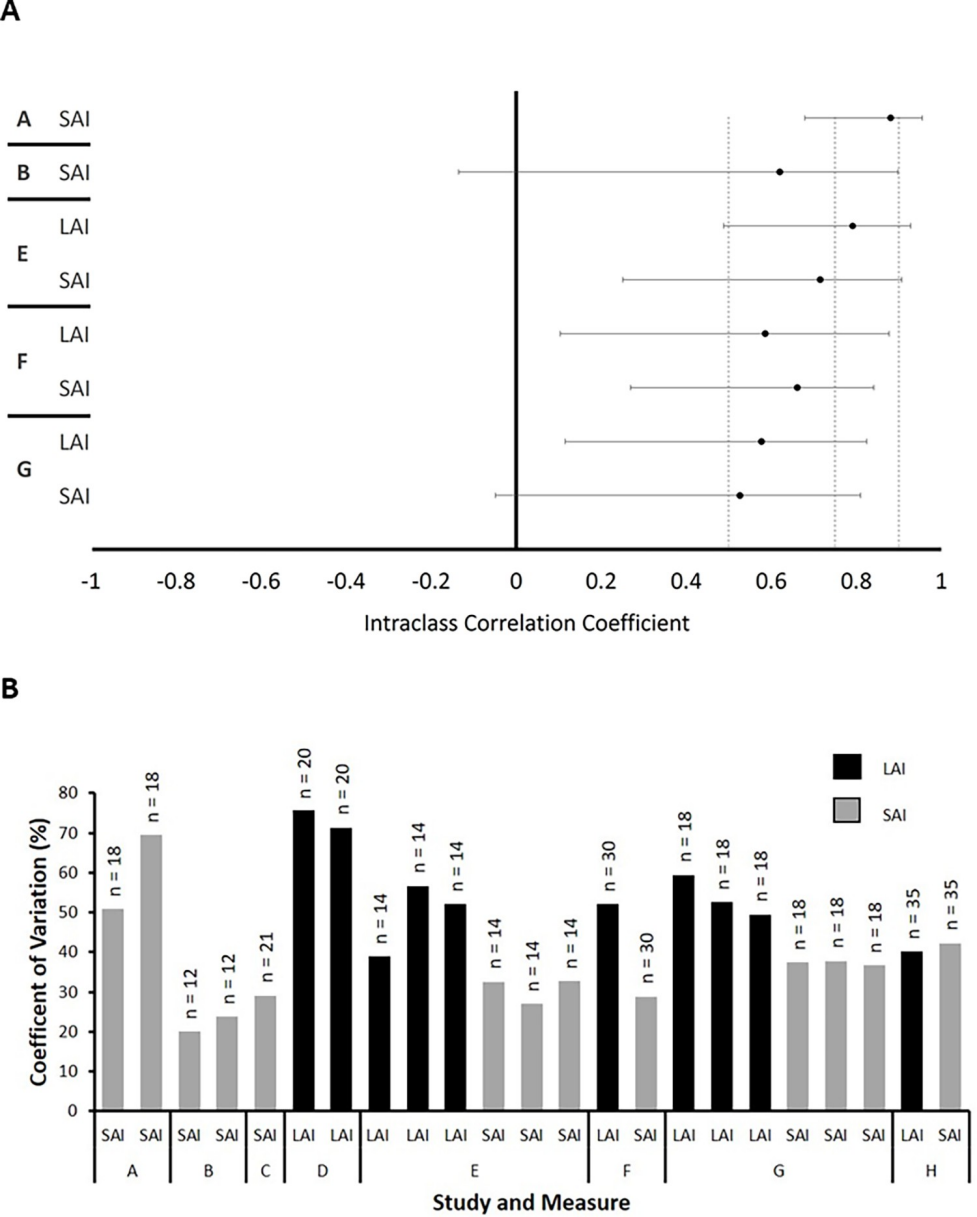
Reliability and heterogeneity measures. Panel A shows the SAI and LAI ICC values with 95% confidence intervals for each study included in dataset 2. Vertical dashed lines reflect the border between low, moderate, strong, and excellent reliability, from left to right. Panel B shows the sample sizes and coefficients of variation, calculated in baseline data from each included study. A = Tsang et al. 2014; B = Tsang et al. 2015; C = Bailey et al. 2016; D = Turco et al. 2017; E = Turco et al. 2018; F = Turco et al. 2019; G = Toepp et al. 2019. H = Harasym et al. 2020.

ICCs shown in Fig 6 indicate the relative reliability of SAI and LAI. Based on the ICC and CIs seen in Fig 6, SAI from study A (ICC = 0.881, 95% CI [0.679, 0.955]) and LAI from study E (ICC = 0.792, 95% CI [0.488, 0.928]) showed strong reliability. SAI from study E (ICC = 0.715, 95% CI [0.251, 0.907]) showed moderate-strong reliability, and SAI from study B (ICC = 0.620, 95% CI [-0.135, 0.898]), study F (ICC = 0.611, 95% CI [0.269, 0.841]) and study G (ICC = 0.526, 95% CI [0.049, 0.810]) showed low-moderate reliability. As shown in Fig 3B, the majority of CVs for SAI fall below 50% (11/13 datasets) and most CVs for LAI exceed 50% (7/10 datasets).

**Fig 7:**
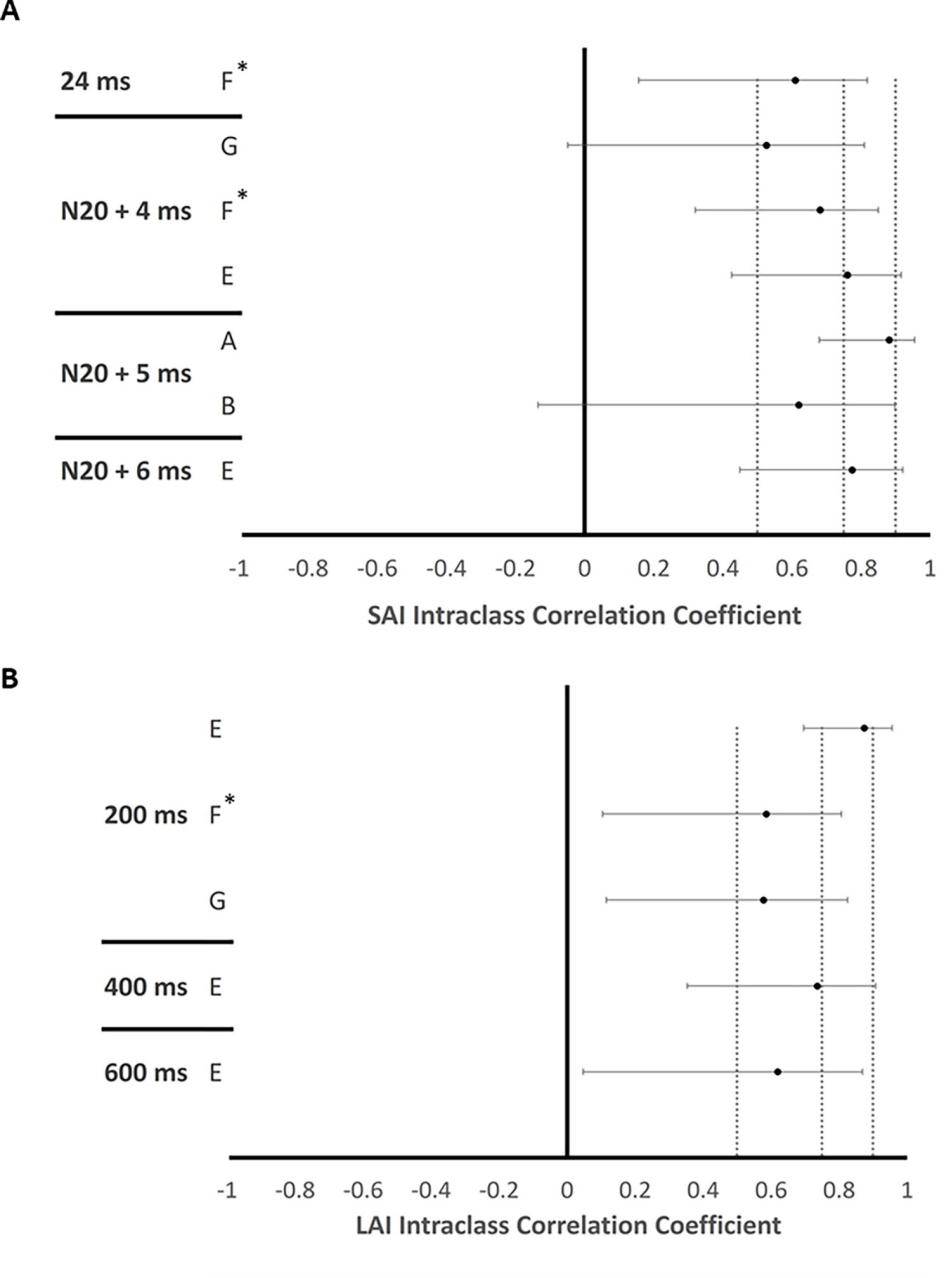
Reliability according to ISI. Panel A and B shows the reliability of measurements performed at each ISI for SAI and LAI, respectively. ICC values with 95% confidence intervals are shown for each ISI tested in the studies that comprise dataset 2. Vertical dashed lines reflect the border between low, moderate, strong, and excellent reliability, from left to right. * Indicates a that the ICC and 95% confidence interval for Study F are referenced from Turco et al., [14]. A = Tsang et al. 2014; B = Tsang et al. 2015; C = Bailey et al. 2016; D = Turco et al. 2017; E = Turco et al. 2018; F = Turco et al. 2019; G = Toepp et al. 2019. H = Harasym et al. 2020.

ICCs shown in Fig 7 indicate the relative reliability of SAI and LAI at the various ISIs used in the included studies. Based on the ICC and CIs seen in Fig 7A, SAI assessed at an ISI of 24ms shows moderate reliability (Study F), SAI at N20+4 ranges from low-to-moderate to strong (Study F, Study G, and Study E), N20+5 shows both low and strong reliability (Study A and Study B), and N20 +6 demonstrates moderate-to-strong reliability (Study E). The ICC values and 95% CIs shown in Fig 7B indicate that the relative reliability of LAI assessed using an ISI of 200ms may be moderate or strong (Study E, Study F, and Study G), LAI assessed at 400ms is moderate-to strong (Study E), and LAI assessed using an ISI of 600ms is low-to-moderate (Study E).

## Discussion

This retrospective study reviewed SAI and LAI records from eight previous studies published by our lab with the aim of understanding the normative range and depth of afferent inhibition in healthy controls. We also sought to quantify the reliability of both measures and identify the prevalence of individuals that do not show inhibition. The depth of SAI and LAI were not associated with age, time of day, or biological sex. Here, we discuss the implications of these findings.

### Interpreting the distribution of SAI and LAI values

The term afferent *inhibition* implies that the expected response in this protocol is inhibition, and any ratios above 1 are therefore considered “atypical”. However, in our data, ~12% and ~7% of healthy controls do not exhibit short- and long-latency afferent *inhibition*, respectively. This suggests that in a healthy control population, SAI and LAI are not expected in all participants. This challenges the inclusion of the term “inhibition” when labelling these measurements. Importantly, labelling the measurement as long- or short-latency afferent “*inhibition*” may influence decisions to include or exclude data and arbitrarily identify individuals as non-responders. For instance, when an individual does not demonstrate inhibition, a researcher may be prompted to re-measure or exclude the data based on the expectation that inhibition should occur. There is, however, no evidence to suggest a biological distinction between individuals that demonstrate inhibition versus facilitation when short- and long-latency ISIs are used. We therefore propose more neutral terms of short- and long-latency afferent “interaction” to characterize these neural circuits and emphasize that only outliers based on predefined statistical criteria should be removed from SAI/LAI datasets.

In our data, SAI values exhibit a mean ratio of 0.69 ± 0.28 (~30% inhibition). If a predefined threshold of 2 times the standard deviation is used to remove outliers, then values between 0.13 to 1.25 should be considered within the typical range. Using the same approach for LAI (mean: 0.60 ± 0.34), the lower and upper thresholds for inclusion are -0.08 and 1.28, respectively. Since a negative ratio has no meaning in this context, LAI ratios > 1.28 are considered outliers. Certainly, outlier detection thresholds will vary depending on the specific dataset, and this is appropriate given the considerable study-to-study differences in SAI and LAI variability reported here (Fig 5B) and by others [15]. In contrast, removing SAI or LAI measurements when the ratio exceeds 1 imposes a fixed exclusion criterion which impacts the data differently depending on the distribution of observations in each study.

No correlation was found between age and the depth of SAI, but we did find that SAI was significantly weaker in Study H, which sampled older individuals (59 ± 3 years), than in study A, which sampled a younger cohort (21 ± 2 years). Specifically, Harasym et al. [22] (i.e., study H) studied a group of post-menopausal women while Tsang et al. [16] (i.e., Study A) studied a sample of young males and females. This finding may support a previous meta-analysis reporting a significant decline in SAI with age [25]. Notably, our correlational analyses were limited to the age range of 18–35 years. Therefore, our analyses suggest that age does not influence afferent inhibition within this range of healthy, young participants but do not speak to the age-related decline in afferent inhibition that may be present beyond 35 years of age.

It is interesting to note that the portion of participants in Study H (11/35) that did not show inhibition was higher than for any of the other included studies. Since between-subject SAI variability for Study H was similar to that in the other studies (Fig 1A), this can be attributed to the mean SAI ratio falling nearer to 1.0 in this group. It important not to interpret this finding as diminishing the usefulness of SAI in the elderly. The border between inhibition and facilitation (CSTS/TS = 1.0) is irrelevant as long as measurements are compared against well documented population norms or appropriate control groups. Previous studies that have compared SAI values measured in the morning versus afternoon/evening have reported no significant differences. Hwang and colleagues [26] found no effect of time of day in healthy elderly controls when SAI was assessed in the morning and four hours later (i.e., in the afternoon). Further, Bocquillon et al [27] assessed SAI in the morning (9:20 to 11:00) and in the evening (18:00–19:30) and found no difference. In agreement with these findings, we did not observe a relationship between testing time and SAI depth (Fig 3B). We also did not observe any relationship between strength of LAI and time of day (Fig 3E). This agrees with an earlier finding that there is no difference between LAI measured in the morning versus in the evening [27].

### Between-session variability and reliability between studies

Our data also indicate that substantial between-session variability exists in LAI and SAI measurements (Fig 4B and 4D). Not only are between-session differences relatively large, at 0.21 and 0.27 for SAI and LAI respectively, but the standard deviation of these differences is considerable. We therefore repeat our previous recommendation against using SAI and LAI as markers of individual neurophysiological change [14]. This echoes prior recommendations against the use of paired-pulse measures for investigating change on the individual level [28]. In the present study, we assessed individuals for whom repeated measurements were available for SAI (n = 92) and LAI (n = 62). Notably, ~17% and ~21% of participants did not exhibit inhibition for SAI and LAI, respectively, on at least one occasion. Visual inspection of Fig 4B and 4D also reveals that it is relatively common for these measures to fluctuate between values above and below 1.0 on subsequent sessions. Notably, only one case where SAI or LAI crossed between facilitation and inhibition fell outside of the range of fluctuation magnitudes when facilitation or inhibition was maintained (Fig 5E). Therefore, the variations in SAI or LAI crossing 1.0 are not atypical when compared to the other fluctuations, suggesting that they can be explained as part of the overall variability inherent in SAI and LAI measurements. We also found that TS intensity was negatively correlated with the size of between-session differences in LAI (Fig 5B). This suggests that higher TS intensities are associated with lower between-session variability. It is unclear why the same relationship was not observed for SAI. It is possible that the TS intensity that maximizes reliability is different between SAI and LAI. Presently, the TS intensities are chosen using the same conventions for SAI and LAI (i.e., 120% of RMT or the TS intensity that evokes a MEP of ~1mV), and this may or may not be appropriate. Future investigations should systematically compare the reliability of LAI and SAI acquired using range of TS intensities (e.g., between 110% and 180% of RMT). Such investigations will be able to identify appropriate TS intensities for each measure that will minimize between-session fluctuations. These data have important implications for future SAI and LAI research, particularly for studies investigating changes over time (i.e., intervention). We suggest implementing sham or time control conditions in all such studies and measuring reliability statistics of smallest detectable change (SDC) and ICC [23, 24, 29]. Also, in studies where SAI or LAI measurements are compared between different populations (e.g., [10, 11, 30, 31]), we recommend that multiple assessments be performed on different days to increase precision and thereby improve the sensitivity.

Relative reliability is assessed in the present study using ICC values. A critical companion to the calculation and assessment of ICC values is the 95% CI. Based on both the ICC and CIs seen in Fig 3, we found that SAI and LAI ranged from low to strong reliability across studies (Median ICC = 0.603). Overall, there is a lack of consistency for the ICC values of both SAI and LAI across the assessed studies. One plausible reason for this variability in reliability may be differences in the experimenters acquiring SAI and LAI. Few studies have investigated the inter-rater reliability of TMS measures [32], and none have investigated SAI or LAI to our knowledge. However, the inter-rater reliability of motor hotspot has been assessed between an experienced and a novice experimenter revealing differences ranging from 2.39 mm to 9.22 mm (mean: 4.89 mm) between raters [33]. Differences in experimenter experience could result in variability in TMS location when different experimenters assess SAI or LAI between each repeated measurement session, and slight differences in the consistency of coil positioning between experimenters may explain some of the ICC variation. The intensity of the TMS pulses also influences the intra- and inter-rater reliability of MEP amplitudes, with pulses delivered at 120% of resting motor threshold (RMT) yielding poorer reliability than 140% of RMT [34]. In our studies, TMS was delivered at the intensity that elicited MEP amplitudes of ~1 mV. This intensity may have varied significantly with respect to individual motor thresholds between participants and between studies.

In addition, the ISI used in paired-pulse measures of intracortical facilitation (ICF) and short-interval intracortical inhibition (SICI) may influence their intra- and inter-rater reliability [34]. This may be relevant to the between-study differences in ICC reported here, since SAI was acquired using the latency of the N20 + 4ms [14, 20, 21], 5ms [16, 17], or 6ms [20] in the surveyed studies (see Table 2). We investigated the reliability of the SAI and LAI when evoked at each of the ISIs used within this group of studies and found no obvious pattern to suggest that a particular ISI improves or hinders reliability. However, whether the choice of ISI contributes significantly to between session variability in these measures in general should be determined in future studies explicitly designed to answer this question.

The coefficient of variation reflects the between subject variability existing within the data from a given study. Because the ICC depends on the between-subject variability [35], the CV may aid in its interpretation. Therefore, differences in the heterogeneity of samples across studies (Fig 5B) may have led to variations in the ICC. Site variability may also result in vastly different ICC values. As presented in the supplementary data published by Brown et al. [15], the relative reliability of SAI differed drastically between sites. Specifically, relative reliability ranged from low (ICC < 0.5) to moderate (ICC < 0.8) to high (ICC > 0.8) at the various testing sites (Appendix A, Supplementary Table 6 by Brown et al [15]). Indeed, our findings of relative reliability may not extend to other groups or even to past studies within our lab which were performed by different experimenters with different sample demographics and slightly different methodologies. Given these various issues, the ICC for SAI and LAI measurements should be considered lab- and study-specific and estimates of relative reliability should be integrated into the design of research studies.

Another plausible cause for the variance in the observed ICCs is differences in the number of trials that are averaged to calculate the average MEP_TS_ and MEP_CSTS_ amplitude. It appears that studies using fewer TS/CSTS trials have lower reliability (Study F: # of CSTS = 12; ICC_SAI_ = 0.661; ICC_LAI_ = 0.586) whereas those with a higher number of TS/CSTS trials have higher reliability (Study E: # of CSTS_MEP_ = 15; ICC_LAI_ = 0.792 and Study A: # of CSTS_MEP_ = 20; ICC_SAI_ = 0.881). An increase in reliability as a function of the number of trials has previously been observed for both single-pulse [36, 37] and paired-pulse TMS assessments [36]. The reliability of both single-pulse and paired-pulse TMS estimates steadily increased before eventually plateauing [36, 37]. Future studies should look to explore the relationship between the number of trials and relative reliability of SAI and LAI further to identify the minimum number of pulses needed to achieve good reliability.

The variations in ICC seen across the studies presented in this analysis as well as in previous work [15] brings to light important caveats to the usage of reliability statistics. While background literature can provide a baseline assessment of a measure’s reliability, relative reliability is inherently a within-study assessment which can differ drastically between studies. As such, we suggest that researchers avoid deferring to previously published reliability statistics in lieu of reporting their own reliability measures. Rather, the published experimental protocols which led to estimates of good reliability (# of pulses; muscle tested; sample size) should be adopted in future studies. Further, due to the variance in reliability, experimenters should, wherever possible, implement reliability statistics alongside their regular analyses. These reliability statistics will aid in the interpretation of the study results, with lower reliability indicating methodological limitations in the study protocol which may be hiding true effects.

Of interest to note is the differences in CV between the measures. As can be seen in Fig 3B, the CVs for LAI (38.98% to 73.45%) are, for the majority, higher than those for SAI (12.67% to 50.91%). The noticeable difference in CV values may be related to the different neural pathways that SAI and LAI traverse. While SAI is thought to be representative of either thalamocortical projections to M1, or the activation of inhibitory interneurons from S1 to M1, the pathway for LAI is less certain [2]. However, given the large ISI at which LAI is evoked (>200 ms) [3], there is a possibility for widespread interaction with many cortical areas (PPC, S2, basal ganglia-thalamocortical loop) to produce the inhibitory effect. This opportunity for the involvement of multiple cortical areas in LAI and not in SAI, may lead to an increased variability in the LAI circuit between individuals.

### Limitations

Several issues limit the scope of questions that can be answered by this retrospective investigation. First, all studies included in our analyses involved only right-handed participants. Future studies should examine differences in SAI/LAI as a function of handedness. Interestingly, when tested in right-handed individuals, SAI elicited in the right FDI is stronger than that elicited in the left FDI, indicating hemispheric asymmetry of the measure [38]. Most of our studies were also conducted using a median nerve stimulation intensity of motor threshold for the APB muscle. This pre-empted the possibility of answering questions about the effect of nerve stimulus intensity on the reliability of SAI and LAI measurements. Importantly, we were also unable to investigate the reliability of SAI or LAI in older healthy individuals because the included multiple-session studies were exclusively comprised of young, healthy samples. This is unfortunate because the potential for clinical utility of SAI and LAI will be dependent on reliability in older populations that tend to be affected by Alzheimer’s disease [5, 39], mild cognitive impairment [40], and Parkinson’s disease [10, 11]. Finally, most of the included studies used fewer CSTS-TS trials than is required to achieve good reliability in other paired-pulse TMS measures such as SICI, and ICF [36]. The effect of trial number on the reliability of SAI and LAI has yet to be empirically tested. This should be a priority for future research to avoid impairing the reproducibility due to low trial number.

## Conclusion

This retrospective analysis of our previously published data provides insight about the expected distribution of LAI and SAI ratios, the reliability in our datasets, and potential sources of variability in these measures as assessed in our laboratory. We found that inhibition did not occur in a considerable number of instances, and because of this, we challenge the use of *inhibition* in the labelling of these measures. Consistent with previous reports, we also found that the relative reliability of both measures varied greatly between studies. Inter-individual differences in SAI and LAI were also not associated with time of day or biological sex.

## Supporting information

S1 File SAI and LAI data. Contains single-session and multiple-session SAI, LAI and other data on which analyses were performed. The sheet labeled *Single-session SAI and LAI* contains the data used in the main analyses. This includes, from left to right, the study from which participants originated, the SAI or LAI measurements from the first instance of testing (averaged across ISIs assessed), biological sex, age and time of day. The sheet titled *Multi-session SAI and LAI* includes, the study of origin and the ISI (individual or averaged), SAI or LAI measurements from sessions 1, 2 and/or 3 as applicable, the mean difference between sessions, and the average TS intensity across all sessions as a percentage of the resting motor threshold (%RMT).
(XLSX)
